# Targeting splicing-derived neoantigens for precision cancer immunotherapy

**DOI:** 10.3389/fimmu.2026.1900283

**Published:** 2026-08-27

**Authors:** Mutian Tang, H. Leighton Grimes, Nathan Salomonis

**Affiliations:** 1 Division of Immunobiology, Cincinnati Children’s Hospital Medical Center, Cincinnati, OH, United States; 2 Division of Biomedical Informatics, Cincinnati Children’s Hospital Medical Center, Cincinnati, OH, United States; 3 Immunology Graduate Program, University of Cincinnati College of Medicine, Cincinnati, OH, United States

**Keywords:** alternative splicing, cancer immunotherapy, immune tolerance, neoantigen, spliceosome

## Abstract

Aberrant pre-mRNA splicing in cancer generates protein sequences that are rare or absent in normal tissues, creating a rich source of tumor-specific neoantigens for immunotherapy. These splicing-derived neoantigens arise through diverse mechanisms, including recurrent somatic mutations in core spliceosome components (*SF3B1*, *SRSF2*, *U2AF1*, and *ZRSR2*), epigenetic derepression of transposable elements that give rise to chimeric exon-TE junctions, and coordinated dysregulation of splicing regulatory networks in cancers lacking spliceosome coding mutations. These processes produce two major classes of immunotherapeutic targets: 1) MHC class I-restricted neopeptides that can be recognized by T-cell-based therapies, and 2) extracellular neoepitopes (ExNeoEpitopes) within transmembrane proteins that are accessible to HLA-independent antibody-based modalities, including monoclonal antibodies (mAbs), bispecific engagers (BiTEs), antibody-drug conjugates (ADCs), and chimeric antigen receptor (CAR)-T or CAR-NK cells. Despite their strong immunogenic potential, effective therapeutic exploitation requires overcoming key immunological barriers, including T-cell exhaustion, impaired antigen presentation through MHC-I downregulation, and suppression within the tumor microenvironment. Recent advances in computational neoantigen prediction, immunopeptidomics, surface proteomics, long-read and single-cell isoform sequencing, and AI-guided therapeutic design are enabling more systematic discovery and validation of splicing-derived targets. This review integrates current understanding of the biological origins, immunological barriers, target classes of splicing neoantigens, and the technologies that enable their advancement in cancer immunotherapy.

## 1 Introduction

Pre-mRNA splicing is an essential and precisely regulated step in eukaryotic gene expression that removes intronic sequences and joins exons to produce mature messenger RNA. In human cells, about 95% of multi-exon genes undergo alternative splicing, generating proteomic diversity that is indispensable for normal development and homeostasis (1). The spliceosome is a macromolecular machine composed of five small nuclear ribonucleoproteins (snRNPs) and hundreds of associated proteins. It executes splicing through a highly coordinated sequence of catalytic steps that depend on specific consensus sequences at the 5’ splice site, branch point, and 3’ splice site. Perturbation of any component of this system, whether caused by somatic mutation, aberrant expression, or epigenetic dysregulation, can lead to widespread alterations in the transcriptome with profound pathological consequences.

Recurrent somatic mutations in core spliceosome components (*SF3B1*, *SRSF2*, *U2AF1*, *ZRSR2*), first identified in myeloid malignancies (2–5), represent the best-characterized source of splicing-derived neoantigens. These mutations often arise early and impose characteristic, clonally inherited splicing programs. Such programs can persist during disease evolution, although the abundance and functional consequences of individual aberrant isoforms remain dependent on discrete malignant cell states, lineage programs, and cellular context (6, 7).

Importantly, spliceosome mutations are not the only origin of splicing-derived neoantigens. Cancer cells commonly exhibit widespread mis-splicing through mutation-independent mechanisms such as epigenetic perturbation of splicing factor regulation, derepression of transposable elements, and coordinated dysregulation of splicing regulatory proteins (8–10). From a therapeutic perspective, the relevant immunogenic target is the aberrant isoform itself rather than the upstream mechanism that generated it. Consequently, diverse routes of mis-splicing can produce neoantigens that are equally amenable to immunotherapeutic targeting.

Splicing-derived neoantigens fall into two major classes with therapeutic potential: 1) T-cell-based strategies that target short peptides derived from aberrant splicing and presented by MHC class I complexes to cytotoxic T lymphocytes; 2) surface-directed approaches that recognize aberrant full-length protein isoforms containing newly created extracellular neoepitopes (ExNeoEpitopes) that can be targeted by antibody-based therapeutics without HLA restriction (11–13). Since these epitopes are not HLA-restricted, a single therapeutic agent can potentially be applied across a broad patient population.

The interplay between RNA splicing and cancer immunotherapy has been the subject of several recent reviews, which have focused primarily on how spliceosome mutations and alternative splicing generate MHC class I-restricted neoantigens for T-cell recognition and on the computational prediction of these epitopes (14–16). Building on this foundation, we discuss both MHC-bound splicing neopeptides and the relatively underexplored extracellular neoepitopes (ExNeoEpitopes) created within transmembrane proteins, highlighting their complementary therapeutic opportunities for T-cell- and antibody-based immunotherapies. We further integrate the technological advances enabling their discovery and validation, including several proteomics-based approaches, long-read and single-cell isoform sequencing, and AI-guided structural and therapeutic design, together with the specificity, tolerance, and safety considerations that shape their clinical translation. By integrating the biological origins of aberrant splicing, both target classes, recent technological advances, and the key challenges facing clinical translation, this review provides an up-to-date perspective on the current state of the field and its future directions.

## 2 Sources of splicing neoantigens

**Figure 1:**
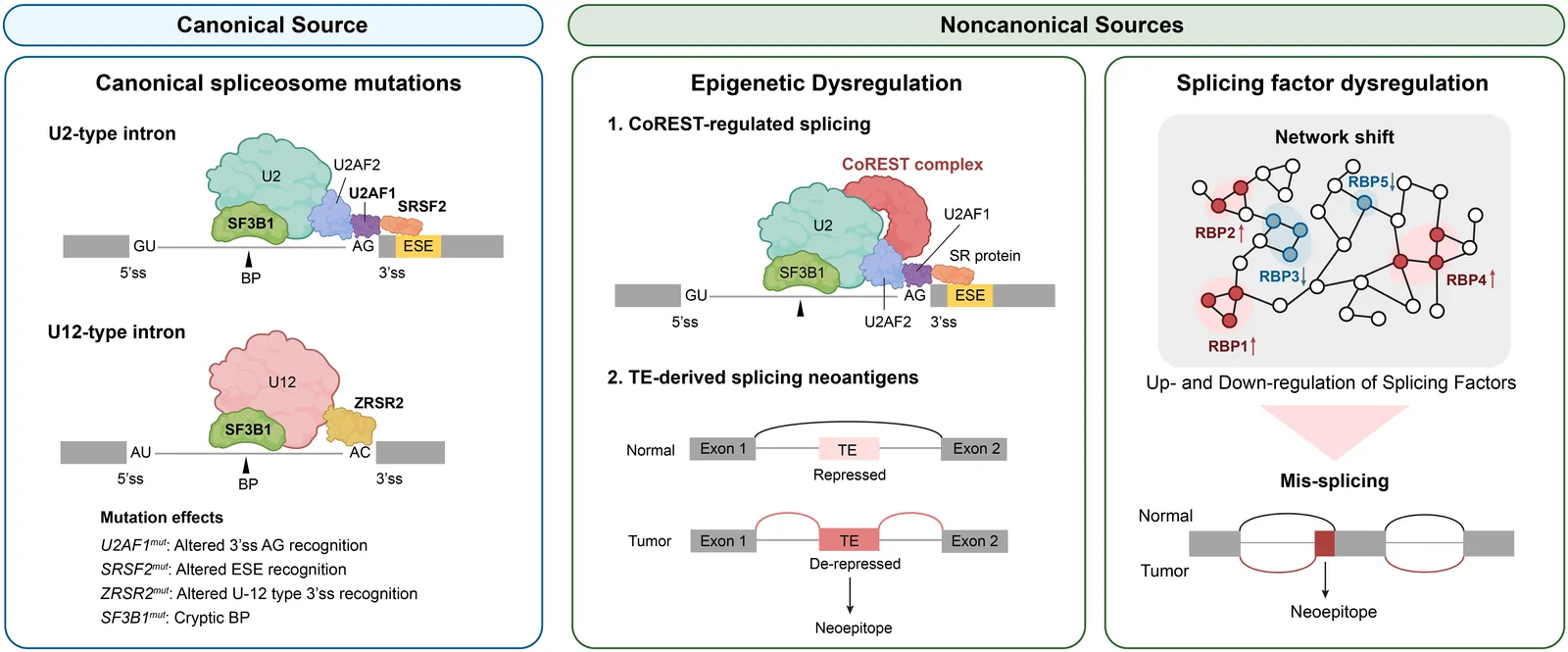
Canonical and noncanonical sources of splicing-derived neoantigens in cancer. Canonical source: The major U2-type spliceosome recognizes GU/AG splice sites via SF3B1, U2AF1, U2AF2, and SRSF2, while the minor U12-type spliceosome processes AU/AC introns via SF3B1 and ZRSR2. Recurrent mutations disrupt splice-site (ss) recognition: *U2AF1* (3’ss AG), *SRSF2* (ESE), *ZRSR2* (U12-type 3’ss), and *SF3B1* (cryptic branch point), producing aberrant transcripts and tumor-restricted neoepitopes. Noncanonical sources: The CoREST repressor complex binds the splicing factors U2AF2 and SRSF1, coupling chromatin regulation to splice-site selection. Epigenetic derepression of transposable elements (TEs) exposes cryptic splice sites, producing tumor-specific junction exon-TE transcripts (JETs). Dysregulation of RNA-binding proteins (RBPs) further rewires the splicing network, generating neojunction-encoded neoepitopes. Created in BioRender. Tang, M. (2026) https://BioRender.com/oucnhi6.

Splicing-derived neoantigens arise when aberrant pre-mRNA processing generates transcript sequences that are rare or absent in normal tissues. The best-characterized source is somatic mutation of core spliceosome components, which produces recurrent, mutation-associated splicing programs that are clonally inherited but variably expressed across discrete cellular states in malignancies that harbor these mutations. However, increasing evidence indicates that noncanonical mechanisms independent of spliceosome gene mutations, including epigenetic deregulation of co-transcriptional splicing (17), transposable element derepression (9, 10), and coordinated splicing factor dysregulation (11, 18), can also generate tumor-specific aberrant junctions across a broader range of cancer types (**Figure 1**).

### 2.1 Canonical source: spliceosome mutations

The spliceosome is a large ribonucleoprotein complex that mediates pre-mRNA splicing by removing introns and joining exons during gene expression (19). Splicing factor mutations are a dominant class (5-30%) of somatic “hotspot” driver mutations or deletions in myeloid neoplasms (2–4). The four most commonly affected genes, *SF3B1*, *SRSF2*, *U2AF1*, and *ZRSR2*, typically occur in a mutually exclusive pattern within individual patients and clones (2). Beyond these four dominant genes, myeloid malignancies also harbor recurrent or rarer mutations and deletions in additional splicing components, including *PRPF8* and *LUC7L2* (20, 21), together with low-frequency lesions in *SF3A1*, *SF1*, *PRPF40B*, and *U2AF2*, each capable of perturbing splice-site or branch-point recognition and thereby broadening the aberrant-junction repertoire available for neoantigen formation (2). Notably, these mutations are also recurrent in other malignancies, including chronic lymphocytic leukemia (CLL), uveal melanoma, and breast cancer (22–24). Each mutant spliceosome factor generates a characteristic repertoire of aberrant splice events, associated with either cryptic exon inclusion, alternative branchpoint usage, intron retention, or altered splice site selection (**Table 1**) (25–29). The resulting aberrant transcripts can produce novel peptide sequences absent from the normal proteome, which makes spliceosome mutations a direct and well-characterized source of splicing-derived neoantigens, particularly in myeloid neoplasms where they are most prevalent (30, 31).

**Table 1:** Spliceosome mutations and their splicing consequences.

Gene	Function in spliceosome	Hotspot mutation(s)	Mechanistic consequence	Representative affected transcripts	Cancers with recurrence	References
*SF3B1*	U2 snRNP; binds branch-point adenosine	K700E, K666N, R625H/C/L, H662Q, E622D	Change-of-function; disrupted SF3B1-SUGP1 interaction drives use of cryptic branch points 10–30 nt upstream of canonical sites, generating aberrant 3’ splice site junctions	*MAP3K7*, *TMEM14C*, *ENOSF1*, *ABCB7*; ~50% of aberrant transcripts subject to NMD	MDS-RS (~65%); CLL; uveal melanoma; breast cancer	(3, 22–25, 29, 117, 118)
*SRSF2*	SR protein; binds ESE motifs via RRM	P95H, P95L, P95R	Change-of-function; shifts ESE binding preference from balanced SSNG recognition (CCNG and GGNG equally) toward preferential CCNG binding, driving cassette exon inclusion/skipping	*EZH2* poison exon (NMD target); *CASP8*; *BCOR*; *FYN*; *HNRNPA2B1*; *CSF3R*	MDS; CMML; AML; DLBCL	(2, 27, 34, 119, 120)
*U2AF1*	35-kDa subunit of U2 auxiliary factor (U2AF); recognizes 3′ splice site AG via CCCH zinc fingers	S34F/Y (ZnF1); Q157P/R (ZnF2)	Change-of-function. S34 mutations alter −3 nucleotide preference (U➔C); Q157 mutations alter +1 preference (A➔G). The two hotspots produce non-overlapping junction sets	Hotspot-specific; S34 and Q157 targets minimally overlap	MDS; AML; CMML; lung adenocarcinoma	(2, 4, 26, 121)
*ZRSR2*	Minor (U12) spliceosome; U12 intron recognition	Loss-of-function across coding region (no hotspots); X-linked	Loss of function; disrupts U12-type intron splicing, causing widespread U12 intron retention while preserving U2-type splicing	U12-intron-containing genes in E2F, MAPK, and other oncogenic pathways	MDS; AML (predominantly male patients)	(2, 28)

Summary of the four recurrent spliceosome gene mutations that generate splicing-derived neoantigens, their mechanistic impact on pre-mRNA splicing, representative affected transcripts, and cancer types in which they recur. Each mutation produces a characteristic and clonally stable aberrant splicing repertoire that serves as the substrate for neoantigen discovery. CMML, chronic myelomonocytic leukemia; DLBCL, diffuse large B-cell lymphoma.

#### 2.1.1 *SF3B1* mutations

*SF3B1* encodes a core component of the U2 snRNP that contacts the branch point adenosine during spliceosome assembly (32). *SF3B1* hotspot mutations (K700E, K666N) act as change-of-function alterations that cause widespread use of cryptic branch points 10–30 nucleotides upstream of the canonical site, which generates transcripts with novel 3’ splice site junctions (25). *SF3B1* mutations are found in about 65% of patients with myelodysplastic syndrome (MDS) with ring sideroblasts (MDS-RS) (3), and are also recurrent in acute myeloid leukemia (AML), CLL, uveal melanoma, and breast cancer (22–24).

#### 2.1.2 *SRSF2* mutations

*SRSF2* encodes a serine/arginine-rich (SR) protein that functions as a splicing enhancer by binding exonic splicing enhancer (ESE) sequences (33). The hotspot mutation P95H alters the RNA-binding specificity of this SR protein, shifting ESE motif preference from balanced CCNG/GGNG motif recognition to preferential CCNG binding (27). This altered sequence preference leads to widespread cassette exon inclusion and skipping events in a characteristic set of targets. Most notably, mutant SRSF2 promotes inclusion of a poison exon in *EZH2*, introducing a premature termination codon (PTC) that triggers nonsense-mediated decay (NMD), while also altering splicing of *CASP8*, *BCOR*, and *FYN* (27, 34, 35).

#### 2.1.3 *U2AF1* mutations

*U2AF1* (also known as *U2AF35*) encodes the 35 kDa subunit of the U2 auxiliary factor (U2AF) complex, which recognizes the AG dinucleotide at the 3’ splice site and is required for initiating spliceosome assembly (36). Hotspot mutations occur at S34 in the first zinc finger and Q157 in the second, and these two mutations differentially alter splice site recognition (4, 26). S34F/Y mutations shift nucleotide preference at the -3 position relative to the AG dinucleotide, disfavoring uridine and favoring cytidine; whereas Q157P/R mutations alter preference at the +1 position immediately after the AG (26). Since S34 and Q157 mutations generate largely distinct aberrant splicing programs rather than convergent ones, each generates a unique repertoire of exon-junction neoepitopes.

#### 2.1.4 *ZRSR2* mutations

*ZRSR2* encodes a component of the minor (U12) spliceosome rather than the major (U2) spliceosome (28). Loss of *ZRSR2* selectively disrupts U12-type intron splicing, which causes widespread intron retention while largely preserving U2-type splicing (28). The affected transcripts include genes in E2F, MAPK, and other oncogenic pathways (28). Unlike other spliceosome genes, *ZRSR2* lacks mutational hotspots, with loss-of-function mutations distributed across the coding region (2). Since *ZRSR2* is X-linked, a single mutation is sufficient for inactivation in males, which explains the male predominance of these mutations (28). Consequently, *ZRSR2*-mutant cells produce a distinct set of neoepitopes derived primarily from U12-type intron retention.

### 2.2 Noncanonical sources of splicing neoantigens

#### 2.2.1 Epigenetic regulation

Epigenetic macromolecular complexes tightly regulate gene expression at the chromatin level and have recently been found to colocalize with RNA splicing machinery during active transcription (37). Fisher et al. identified unique interactions between the CoREST repressor complex (LSD1-HDAC1-CoREST) and core components of the RNA splicing machinery, demonstrating direct physical binding between CoREST complex components and the splicing factors U2AF2 and SRSF1 (17). Using mass spectrometry, *in vivo* binding assays, and cryo-electron microscopy (cryo-EM), the authors provided direct structural evidence for the interaction between the RRM2 domain of U2AF2 and the LSD1-RCOR1 complex (17).

Pharmacological disruption of these interactions using the CoREST-selective inhibitor corin drove extensive splicing reprogramming across melanoma and other malignancies (17). Such reprogramming resulted in the *de novo* generation of bona fide splicing-derived neoantigens that enhanced tumor immunity (17). This establishes a mechanistic link between chromatin state and splicing neoantigen production that operates independently of spliceosome gene mutations.

#### 2.2.2 Transposable elements

Transposable elements (TEs) account for nearly half of the human genome and are normally epigenetically silenced in somatic tissues through DNA methylation and repressive histone modifications. In cancer, however, global epigenetic remodeling frequently leads to DNA hypomethylation and altered chromatin states that derepress TE sequences (38). Upon reactivation, TEs can expose cryptic splice donor and acceptor sites embedded within their sequences. When these TE-derived splice sites occur within or adjacent to protein-coding genes, they may be recognized by the spliceosome and incorporated into splicing reactions with neighboring exons. This generates noncanonical exon-TE chimeric transcripts, commonly termed junction exon-TE transcripts (JETs) (9, 10). Since TEs remain silenced in most normal tissues, these exon-TE junctions are largely tumor-specific. Translation of such transcripts can therefore produce novel peptide sequences absent from the normal proteome, establishing TE-driven mis-splicing as a distinct and epigenetically regulated source of splicing-derived neoantigens.

Two complementary studies provided direct evidence that JETs generate immunogenic targets. In non-small cell lung cancer, Merlotti et al. identified tumor-specific JETs encoding peptides presented on HLA class I molecules and recognized by autologous patient T cells (9). In parallel, Burbage et al. validated the presentation of JET-derived peptides on MHC class I in murine tumor models and demonstrated that vaccination against these peptides delayed tumor growth (10). Critically, Burbage et al. showed that inactivation of the TE-silencing methyltransferase *Setdb1* induced new immunogenic JETs (10). These findings suggest that pharmacological modulation of DNA methylation can expand the JET-derived neoantigen repertoire. Given that epigenetic modulators are most widely used in hematologic malignancies, this mechanism may be particularly relevant in these diseases.

#### 2.2.3 Coordinated, mutation-independent splicing dysregulation

Beyond spliceosome mutations and TE-derived junctions, computational evidence suggests that coordinated splicing factor dysregulation during malignant transformation may represent an additional source of splicing neoantigens, even in cancers lacking spliceosome gene mutations. Li et al. applied SNAF (Splicing Neoantigen Finder) to a TCGA melanoma cohort, a cancer type that does not typically harbor spliceosome mutations, and found that splicing neoantigen burden correlated with coordinated changes in splicing factor expression rather than with individual gene mutations, with the RNA-SPRINT module identifying regulatory networks of mis-splicing that predicted patient survival and immunotherapy response (11).

Direct experimental support for this mechanism was provided by Kwok et al., who identified public neoantigens derived from aberrant splicing of *GNAS* and *RPL22* across diverse cancer types including glioma, mesothelioma, prostate cancer, and liver cancer, none of which carry recurrent spliceosome mutations (18). These neojunction-derived neoantigens were endogenously generated and presented on MHC class I under physiologic conditions, and T-cell receptor (TCR)-engineered CD8+ T cells specific for these neoantigens mediated tumor cell eradication. Critically, using CRISPRi and siRNA knockdown of splicing-related genes including *CELF2*, *SNRPD2*, and *SF3A3*, the authors demonstrated that cancer-specific dysregulation of splicing factor expression directly governs neojunction production. This evidence shows that altered expression of splicing factors can generate immunogenic neoantigens in the absence of spliceosome coding mutations (18).

### 2.3 Escape from nonsense-mediated decay

Many mis-spliced transcripts introduce a premature termination codon (PTC) in an NMD-eliciting context. Because NMD is coupled to translation, these transcripts may still generate short-lived translation products capable of contributing to MHC-I peptide presentation before transcript degradation (39). However, NMD reduces steady-state transcript abundance and strongly limits production of stable full-length proteins. Consequently, escape from or avoidance of NMD is particularly important for surface extracellular neoepitopes (ExNeoEpitopes), which require sufficient transcript stability and translation to produce full-length proteins that fold correctly, traffic through the secretory pathway, and localize to the cell surface.

NMD filtering is incomplete for several reasons. Canonical NMD rules predict escape when a PTC resides in the last exon, within approximately the final 50 nucleotides of the penultimate exon, or near the translation initiation site, meaning that many transcripts containing aberrant junctions are not expected to undergo NMD in the first place (40). Even among transcripts predicted to be NMD substrates, a substantial proportion escapes degradation for reasons that remain incompletely understood (40). Moreover, NMD efficiency varies considerably between tissues, individuals, and tumors, and is frequently dysregulated in cancer. For example, recurrent copy-number gains of the NMD regulators *SMG5* and *SMG7* can alter NMD efficiency, leading to tumor-specific differences in the proportion of aberrant transcripts that escape degradation (41). Importantly, NMD-escaping transcripts can be translated and presented as neoantigens. In *SF3B1*-mutant uveal melanoma, Bigot et al. showed that some aberrant junctions were NMD-sensitive whereas others remained stable, and they directly detected aberrantly spliced NET1 protein by targeted mass spectrometry (42). Similarly, polysome profiling of *SF3B1*-mutant cells identified a subset of aberrant transcripts undergoing active translation (43).

NMD can also be therapeutically modulated. Splicing modulators that increase the repertoire of aberrant splice junctions simultaneously suppress NMD through downregulation of the SMG1 kinase, thereby stabilizing PTC-containing transcripts and expanding the pool of presentable neoepitopes (44). Because NMD susceptibility depends strongly on full-length transcript architecture, long-read isoform sequencing (Section 5.3) can improve prediction of whether aberrant transcripts are likely to elicit or evade NMD. Collectively, these findings indicate that although NMD limits the splicing neoantigen repertoire, its efficiency is context-dependent and many aberrant transcripts escape degradation, providing a reproducible and therapeutically expandable source of targetable neoantigens. However, direct measurements of transcript stability, ribosome occupancy, protein production, and antigen presentation or surface localization remain necessary to establish productive expression.

## 3 Immunological tolerance and regulation

The presence of a tumor-specific splicing-derived neoantigen does not necessarily translate into effective immune recognition. Multiple layers of immune tolerance and active suppression within the tumor microenvironment (TME) can limit endogenous responses to these targets. Understanding these barriers is therefore critical for the rational design of neoantigen-directed therapies. This section outlines the major immunological constraints and potential strategies to overcome them.

### 3.1 Tumor specificity and tolerance constraints unique to splicing neoantigens

Unlike neoantigens derived from somatic point mutations, which are strictly absent from the normal genome, splicing-derived neoantigens arise from a physiological process that also occurs at low frequency in healthy tissues. Alternative splicing is highly diverse across normal tissues, and long-read sequencing has revealed thousands of previously unannotated isoforms in healthy samples (45). As a result, a given cryptic junction is frequently tumor-enriched rather than tumor-exclusive, which makes tumor specificity more difficult to establish than for mutation-derived neoantigens.

This distinction also has implications for immune tolerance. Medullary thymic epithelial cells exhibit promiscuous gene expression with extensive splicing diversity, including low-level novel isoforms (46). Accordingly, splicing-derived neoantigens expressed at low levels in the thymus or peripheral tissues may induce central or peripheral tolerance, reducing the endogenous T-cell repertoire (46). To address this challenge, current discovery pipelines prioritize quantitative measures of tumor specificity rather than binary presence-or-absence criteria. For example, Kwok et al. defined cancer-specific neojunctions using tumor and normal positive sample rate thresholds, while SNAF models residual normal expression as a continuous tumor-specificity score (Section 5.1) (11, 18).

### 3.2 General immune barriers to splicing-neoantigen responses

Beyond these splicing-specific considerations, splicing-derived neoantigens encounter the same immune barriers that constrain antitumor immunity more broadly. Immunosuppressive cells and cytokines within the tumor microenvironment can suppress effective T-cell responses even when neoantigens are successfully recognized (47–49). Antigen presentation is also frequently impaired through loss of MHC-I expression or defects in the antigen-processing machinery (50, 51). This barrier primarily affects MHC-restricted splicing neopeptides, whereas antibody-targeted surface ExNeoEpitopes bypass the requirement for peptide-MHC presentation.

Sustained antigen exposure also drives progressive T-cell exhaustion, marked by PD-1, TIM-3, LAG-3, and TIGIT upregulation and loss of cytolytic function (52). This may be particularly relevant for splicing-derived neoantigens in myeloid malignancies, where spliceosome mutations often arise early during clonal evolution, exposing reactive T cells to prolonged antigen stimulation (53). Consistent with this, *SRSF2*-associated splicing-neoantigen-reactive CD8^+^ T cells are detectable in patients but display impaired cytotoxicity that can be restored in healthy donor-derived counterparts (31). Together, these mechanisms help explain why splicing-derived neoantigens are frequently immunogenic yet incompletely eliminated by endogenous immunity, which provides the rationale for the engineered and combination immunotherapies discussed in the following section.

### 3.3 Strategies to overcome immune barriers

Addressing these limitations requires therapeutic strategies that can both restore T-cell functionality and enhance the immunogenic landscape shaped by aberrant splicing. One approach is the adoptive transfer of TCR-engineered T cells derived from healthy donors, which provides a direct means of bypassing endogenous T-cell exhaustion. Kim et al. demonstrated that T cells engineered with TCRs recognizing *SRSF2* mutation-associated neoantigens from healthy donors mediated robust anti-leukemic cytotoxicity in xenograft models, overcoming the functional impairment observed in patient-derived neoantigen-reactive T cells (31). A complementary strategy involves pharmacologic modulation of splicing to increase neoantigen availability and improve immune recognition. Lu et al. showed that splicing modulators such as indisulam and the PRMT inhibitor MS-023 induce the generation of abundant MHC-I-presented neoepitopes with higher immunogenicity rates than conventional mutation-derived neoantigens (**Table 2**). Importantly, combining splicing modulation with PD-1 blockade significantly enhanced tumor control in a T cell- and MHC-I-dependent manner (54). In addition, spliceosome-targeted therapies can engage innate immune sensing pathways. For example, in spliceosome-mutant cells, accumulation of double-stranded RNA activates RIG-I and MDA5 signaling, leading to type I interferon production and amplification of both innate and adaptive anti-tumor responses, as demonstrated in triple-negative breast cancer models (55).

**Table 2:** Representative therapeutics and clinical trials targeting or exploiting alternatively spliced antigens.

Target (antigen/junction)	Splicing origin	Modality/agent	Disease	Stage and key results	Principal challenge/limitation	Ref.
CD44v6 (variant exon 6)	Alternative splicing (variant-exon inclusion)	ADC: bivatuzumab mertansine (BIWI 1)	HNSCC (R/M); esophageal SCC; breast (R/M)	Phase I, HNSCC (NCT02254018): 3 PR/31 patients, MTD 300 mg/m². Phase I, HNSCC or esophageal SCC (NCT02254044): terminated after fatal toxic epidermal necrolysis. Phase I, metastatic breast (NCT02254005, n = 24): no responses, 50% stable disease, MTD not reached. Phase I, R/M breast (NCT02254031, n = 8): terminated with program discontinuation.	On-target/off-tumor skin toxicity (CD44v6 on keratinocytes); fatal toxic epidermal necrolysis; program discontinued	(122–124)
EDB(+) fibronectin (extra domain B)	Alternative splicing (extra-domain inclusion)	ADC: PYX-201 (auristatin payload); +/- checkpoint blockade	Multiple solid tumors	Preclinical ADC eradicates tumors, with enhanced efficacy when combined with anti-PD-1; phase I ongoing (NCT05720117)	Stromal/ECM (not tumor-cell) target; on-target normal-tissue exposure; clinical durability being defined	(62)
*SRSF2*- and *ZRSR2*-induced neojunctions (e.g. *CLK3*, *RHOT2*)	Spliceosome-mutation-derived neojunction (MHC-I)	TCR-engineered T cells (preclinical)	Myeloid neoplasms (AML/MDS)	Public HLA-I neoantigens validated by immunopeptidomics; engineered TCR-T specifically lysed *SRSF2*-mutant leukemia	HLA restriction; endogenous neoantigen-reactive T cells show impaired cytotoxic function	(31)
*GNAS*, *RPL22* and other public neojunctions	Splicing dysregulation, pan-cancer (MHC-I)	TCR-engineered T cells (preclinical)	Glioma, mesothelioma, prostate, HCC and others	Tumor-wide expression; endogenously presented on MHC-I; TCR-T eradicated tumor cells (HLA-dependent)	HLA restriction; endogenous immunity fails to clear; strict tumor-specificity thresholds required	(18)
Splicing-modulator-induced neoepitopes	Pharmacologically induced mis-splicing (MHC-I)	Splicing modulator (e.g. indisulam) + anti-PD-1	Solid tumors (preclinical)	Modulation of splicing enhances anti-tumor immunity; combination improves tumor control (T-cell/MHC-I dependent)	Risk of splicing disruption in normal cells; immune-related adverse events with checkpoint blockade	(54)
SF3b complex (spliceosome)	Direct spliceosome modulation	Small molecule: H3B-8800	SF-mutant MDS, AML, CMML	Phase I (NCT02841540); 84 patients; no objective CR/PR; reduced transfusion requirements in ~14% of patients	Narrow therapeutic window; target engagement without objective clinical responses	(125)

Summary of antibody-based, T-cell-based, and small-molecule agents relevant to alternatively spliced surface antigens and splicing-derived neoantigens, with development stage, key reported outcomes, and the principal challenge or limitation for each. Antibody- and T-cell-based agents are directed at specific alternatively spliced surface antigens or neojunction-derived neoantigens, whereas splicing modulators act by globally reshaping the splicing landscape to expand the presentable neoantigen pool. CR, complete response; ECM, extracellular matrix; HCC, hepatocellular carcinoma; HNSCC, head and neck squamous cell carcinoma; MTD, maximum tolerated dose; PR, partial response; R/M, recurrent/metastatic.

## 4 Two classes of splicing-derived immunotherapeutic targets

**Figure 2:**
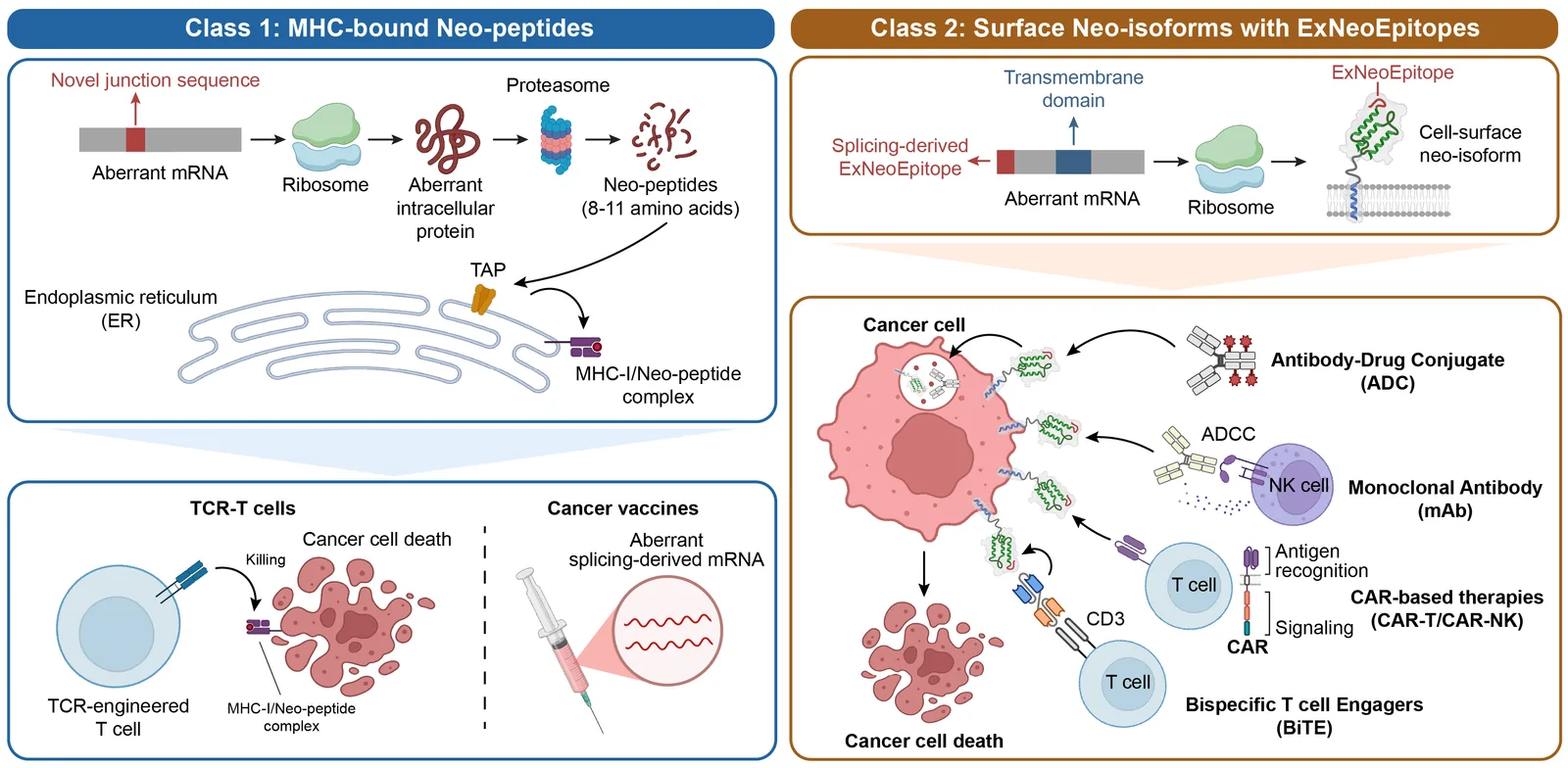
Therapeutic targeting of splicing-derived neoantigens via MHC-restricted and surface-displayed presentation. Class 1: MHC-bound neopeptides (left). Aberrant mRNAs containing novel junction sequences are translated by ribosomes into aberrant intracellular proteins, which are processed by the proteasome into 8–11 amino acid neopeptides. Peptides are transported into the endoplasmic reticulum (ER) via transporter associated with antigen processing (TAP) and loaded onto MHC-I for surface presentation as MHC-I/neopeptide complexes. These complexes are exploited therapeutically by TCR-engineered T cells, which recognize and kill cancer cells displaying the cognate pMHC, and by cancer vaccines that deliver aberrant mRNA to prime endogenous splicing neoantigen-specific T cell responses. Class 2: Surface neo-isoforms with ExNeoEpitopes (right). When aberrant mRNAs encode proteins with intact transmembrane domains, splicing-derived ExNeoEpitopes are displayed extracellularly without MHC restriction. Surface-accessible ExNeoEpitopes serve as targets for multiple antibody-based and cell-based modalities: antibody-drug conjugates (ADCs) deliver cytotoxic payloads, monoclonal antibodies (mAbs) engage NK cells via antibody-dependent cellular cytotoxicity (ADCC), CAR-T and CAR-NK cells recognize the epitope through engineered chimeric antigen receptors coupling antigen recognition to intracellular signaling, and bispecific T cell engagers (BiTEs) bridge CD3 on T cells to the ExNeoEpitope, converging on cancer cell death. Created in BioRender. Tang, M. (2026) https://BioRender.com/oucnhi6.

Splicing-derived neoantigens give rise to two principal classes of immunotherapeutic targets, distinguished by their subcellular localization and mode of immune recognition (**Figure 2**). The first class consists of short intracellular peptides processed by the proteasome and presented on MHC class I for recognition by CD8+ T cells. The second class comprises neo-isoforms of transmembrane proteins displaying novel extracellular neoepitopes (ExNeoEpitopes) on the tumor cell surface, accessible to antibody- and CAR-based modalities independently of HLA restriction.

### 4.1 T-cell-based therapy (class 1): MHC-bound neopeptides

The most extensively characterized class of splicing-derived neoantigens consists of short peptides (8–11 amino acids) generated from aberrant transcript sequences that are degraded through the proteasomal pathway and presented on MHC class I complexes on the tumor cell surface. Unlike point mutation-derived neoantigens, which usually differ from the wild-type peptide by a single amino acid, splicing-derived neopeptides often contain entirely novel sequences created by aberrant splice junctions, cryptic exons, or retained intronic reading frames. Their limited similarity to the normal proteome likely contributes to their high immunogenicity. Consistent with this, Lu et al. reported that 28-43% of splicing-derived peptides generated by pharmacologic spliceosome modulation were immunogenic, compared with less than 10% for conventional mutation-derived neoantigens (54, 56, 57). The increased immunogenicity of splicing-derived peptides likely reflects their greater sequence tumor-specificity, which reduces the likelihood of central T-cell tolerance compared with peptides containing single-residue substitutions.

These neoantigens can also be shared across patients. In a pan-cancer study, Kwok et al. developed a pipeline to identify neojunctions expressed uniformly within tumors and recurrently across patients, and showed that TCR-engineered CD8+ T cells targeting *GNAS*- and *RPL22*-derived neojunction peptides eradicated tumor cells in an HLA-dependent manner (18). The *GNAS* neojunction was expressed tumor-wide across glioma, mesothelioma, prostate cancer, and hepatocellular carcinoma (18).

Spliceosome mutation-derived neoantigens are similarly actionable in myeloid malignancies. Biernacki et al. showed that *U2AF1* Q157R generates MHC class I-restricted neopeptides that are recognized by T cells from healthy donors, and that T cells engineered with a *U2AF1*^Q157R^-specific TCR selectively killed mutant AML blasts while sparing wild-type cells (30). Kim et al. subsequently provided the most comprehensive catalog to date, identifying bona fide neoantigens from stereotyped splicing alterations induced by *SRSF2* and *ZRSR2* mutations and isolating cognate TCRs from healthy donors, patients with active disease, and patients after allogeneic stem cell transplant (31). TCRs recognizing *SRSF2* mutant-induced neoantigens in *CLK3* and *RHOT2* conferred specific cytotoxicity against *SRSF2*-mutant leukemia, and in an AML xenograft model anti-CLK3 TCR T cells reduced tumor burden *in vivo* (31). Together, these studies establish recurrent RNA mis-splicing events as sources of actionable public neoantigens and provide direct proof-of-concept for TCR-engineered T cell therapy against splicing-derived targets (**Table 2**).

### 4.2 Surface-directed therapy (class 2): ExNeoEpitopes for antibody- and CAR-based targeting

Aberrant splicing can remodel the extracellular domains of transmembrane proteins, generating novel peptide sequences at splice junctions that are displayed on the tumor cell surface while remaining absent or minimally expressed in normal tissues. Here, we refer to such tumor-specific extracellular sequences in novel cancer isoforms as extracellular neoepitopes (ExNeoEpitopes), following the nomenclature introduced in the SNAF framework (Section 5.1) (11). Such epitopes have also been referred to as extracellular local splicing variants (ectoLSVs) (58).

The therapeutic potential of alternatively spliced surface antigens is supported by clinically relevant precedents. CD44v6, generated through inclusion of variant exon 6, is overexpressed in many squamous cell carcinomas with limited normal-tissue expression and has been targeted with both monoclonal antibodies and CAR-T cells (59, 60). Similarly, the extra domain B (EDB) splice variant of fibronectin, which is enriched in tumor stroma and vasculature but largely absent from normal adult tissues, has been targeted by CAR-T cells and by an antibody-drug conjugate currently in phase I clinical evaluation (61–63). EDB-directed CAR-T cells demonstrated potent antitumor activity in xenograft models of glioma, lung, and breast cancer (63), and an EDB-specific antibody-drug conjugate (PYX-201) has entered phase I clinical evaluation for solid tumors (NCT05720117) (62). These examples, summarized in **Table 2**, demonstrate the clinical feasibility of targeting alternatively spliced extracellular domains.

ExNeoEpitopes are compatible with multiple therapeutic modalities, including monoclonal antibodies (mAbs) that mediate antibody-dependent cellular cytotoxicity (ADCC) or complement-dependent cytotoxicity (CDC), bispecific T cell engagers (BiTEs) that redirect T cells toward tumor-specific epitopes, antibody-drug conjugates (ADCs), and engineered cell therapies such as chimeric antigen receptor (CAR)-T or CAR-NK cells expressing receptors specific to ExNeoEpitopes. Unlike MHC-restricted neoantigens, ExNeoEpitopes are HLA independent, enabling the same antibody or CAR construct to be applied across patients sharing the same splicing event regardless of HLA haplotype. Their restricted expression in normal tissues also provides a wider therapeutic window than conventional surface antigens, making ExNeoEpitope-targeted therapies a promising complement to T-cell-based immunotherapy.

## 5 Technologies enabling isoform-mediated therapy discovery

Translating splicing-derived neoantigen biology into therapeutic candidates requires computational tools for systematic neoantigen prediction from RNA-seq data, experimental platforms for validating candidate targets at the protein and immune-recognition levels, emerging technologies that resolve full-length isoform structure at single-cell resolution, and AI-based structural modeling for *de novo* therapeutic binder design. This section reviews each of these technologies and identifies key gaps that constrain clinical translation.

### 5.1 Computational frameworks for splicing neoantigen prediction

The identification of splicing-derived neoantigens from RNA-seq data relies on specialized computational pipelines that systematically detect aberrant splice junctions, translate junction-spanning sequences into candidate peptides, and evaluate their MHC binding, immunogenicity, surface accessibility, and tumor specificity. A growing number of frameworks have been developed to address this task, each incorporating distinct algorithmic strategies and analytical strengths (**Table 3**).

**Table 3:** Computational frameworks for splicing-derived neoantigen prediction.

Tool	Input	Target class	Key features	Normal reference	Ref.
End-to-end splicing-neoantigen pipelines					
SNAF	Bulk RNA-seq; HLA type	pMHC + ExNeoEpitope	Junction detection, in silico translation, NetMHCpan/MHCflurry binding, DeepImmuno immunogenicity; SNAF-B reconstructs surface ExNeoEpitopes	GTEx; BayesTS continuous specificity	(11)
NeoSplice	Tumor + matched normal RNA-seq	pMHC	k-mer detection; splice-graph reconstruction; MHC-I binding; immunopeptidomics support	Matched normal	(68)
IRIS	RNA-seq (PSI)	pMHC	Modular AS-target discovery; user-defined references; epitope prediction; screens CAR-T/TCR targets	GTEx + TCGA panel (user-defined)	(69)
ASNEO	RNA-seq	pMHC	Early AS-neoantigen tool; junction-derived peptides; MHC-I binding and immunogenicity scoring	GTEx + UCSC reference	(70)
SpliceMutr	Tumor + normal RNA-seq	pMHC	Differential intron usage; per-gene antigenicity score; MHC-binding prediction; pan-cancer (TCGA)	Differential tumor-vs-normal analysis (unmatched groups)	(71)
ISOTOPE	RNA-seq (+ MHC-I MS)	pMHC	Isoform-guided epitope enumeration across event types; MHC-I binding; self-epitope loss; MS validation	GTEx/tissue-matched normals	(72)
Mutation-anchored tools					
splice2neo	Somatic variants + RNA-seq	pMHC (candidate peptides)	Integrates SpliceAI/MMSplice with LeafCutter/SplAdder; annotates junction peptides; relies on external MHC prediction	Annotation-based	(73)
Regtools	DNA + RNA-seq	Variant-level	Identifies splice-associated somatic variants across pan-cancer cohorts; upstream of neoantigen calling	—	(74)
Upstream/component tools reused by the above (junction or variant-effect detection)					
SplAdder	RNA-seq	—	Graph-based detection, quantification, and testing of alternative splicing events	—	(75)
LeafCutter	RNA-seq	—	Annotation-free quantification of variable intron usage; complex splicing events	—	(76)
General-purpose neoantigen suites with splicing modules					
pVACtools	Somatic variants (VCF)	pMHC	Unified variant-to-vaccine workflow; dedicated fusion (pVACfuse) and splice-site (pVACsplice) modules	—	(77)

Representative tools for identifying splicing-derived neoantigen candidates from sequencing data, grouped by workflow scope. Tools differ in input, target class coverage (pMHC vs ExNeoEpitopes), algorithmic strategy, and normal-tissue reference. Dashes indicate features not applicable to the scope of a tool (e.g., upstream detection tools that do not perform neoantigen or MHC prediction). PSI, percent spliced-in; VCF, variant call format.

#### 5.1.1 End-to-end splicing-neoantigen pipelines

Several frameworks span the full workflow from junction detection to neoantigen prioritization. SNAF (Splicing Neoantigen Finder) is unusual among current frameworks in supporting both pMHC-neopeptide and surface ExNeoEpitope discovery from bulk RNA-seq data (11). The SNAF-T module identifies aberrant exon-exon and exon-intron junctions, performs in silico translation, predicts MHC-I binding affinity (via NetMHCpan or MHCflurry) (64, 65), and applies the DeepImmuno deep learning model to predict immunogenicity (11, 66). To prioritize tumor-specific events, SNAF integrates BayesTS, a hierarchical Bayesian measure of tumor specificity against normal references such as GTEx, and RNA-SPRINT, which infers splicing-factor regulatory networks (11, 67). The SNAF-B module reconstructs full-length transmembrane isoforms to identify extracellular neoepitopes (ExNeoEpitopes), with optional long-read validation (11).

Other pipelines are MHC-I-focused: NeoSplice uses a k-mer approach to detect tumor-specific sequences, projects them onto splice graphs to reconstruct variant transcripts, and predicts MHC-I binding, with immunopeptidomics-based validation (68). IRIS provides a modular pipeline that supports customized workflows for identifying immunotherapy targets from pre-mRNA alternative splicing, with flexible integration of user-defined normal tissue references and epitope prediction (69). ASNEO was among the first RNA-seq tools for alternative-splicing neoantigens, reporting higher immunogenicity scores for splicing-derived than mutation-derived neopeptides in immunotherapy-treated melanoma (70). SpliceMutr identifies tumor-specific antigenic splice variants, predicts MHC-binding affinity, and computes per-gene antigenicity scores across TCGA (71), while ISOTOPE enumerates tumor-specific splicing alterations and their candidate MHC-I neoepitopes, incorporating MHC-I mass spectrometry (72).

#### 5.1.2 Mutation-guided tools

A complementary set of tools begins from somatic variants rather than junction detection alone. Splice2neo, a modular R package, identifies splice junctions arising from somatic mutations by integrating mutation-effect predictors (SpliceAI, MMSplice) with junction-detection tools (LeafCutter, SplAdder) and annotates the resulting peptide sequences; it generates candidate junction peptides but relies on a downstream tool for MHC-binding prediction (73). Applied to melanoma cohorts, splice2neo identified an average of 1.7 tumor-specific splice junctions per patient with a false discovery rate below 5%, and these splicing-derived neoepitope candidates showed lower self-similarity to wild-type peptides than point mutation-derived candidates (73). Regtools complements these by providing a framework for integrating DNA and RNA sequencing data to identify splice-associated somatic variants across pan-cancer cohorts (74).

#### 5.1.3 Upstream and general-purpose components

The pipelines above build on general splicing-analysis tools that are not themselves neoantigen callers. Graph-based methods such as SplAdder detect, quantify, and test aberrant splice junctions (75), while intron-centric methods such as LeafCutter quantify variable intron usage without transcript annotation (76). These tools provide the aberrant junctions that downstream neoantigen pipelines translate and evaluate. Separately, general-purpose neoantigen suites have added splicing modules: pVACtools couples somatic-variant (VCF) input with dedicated components for fusion (pVACfuse) and splice-site-derived (pVACsplice) neoantigens within a unified variant-to-vaccine workflow (77).

Collectively, these tools have expanded the computational landscape for splicing neoantigen discovery. However, direct comparison remains difficult because no standardized benchmark exists and tools differ in input requirements, supported splicing-event types, normal-tissue references, and immunogenicity prediction. Common limitations include tumor-specificity assessment, false-positive junction predictions, and limited experimental validation.

### 5.2 Immunopeptidomics and proteogenomic validation immunopeptidomics

#### 5.2.1 Immunopeptidomics

Mass spectrometry (MS)-based immunopeptidomics provides direct evidence of antigen presentation by isolating HLA-peptide complexes from tumor cells or tissues, followed by liquid chromatography-tandem mass spectrometry (LC-MS/MS) analysis against proteogenomic databases that incorporate aberrant splice junction-derived sequences (78). Using this approach, multiple studies have confirmed that splicing-derived peptides are processed and presented on MHC-I. Validation of SNAF-T predictions in ovarian cancer and melanoma demonstrated that junction-derived neopeptides can be detected by spike-in MS/MS, supporting their endogenous presentation (11). Similarly, incorporation of retained intronic sequences into proteogenomic databases enabled the identification of intron retention-derived neoepitopes presented on MHC-I (12). Integration of immunopeptidomics with ribosome profiling and RNA-seq further revealed that thousands of noncanonical peptides, including those arising from aberrant splicing, are actively translated and presented (79).

Efforts to scale neoepitope-HLA discovery have further advanced the field. A high-throughput platform combining *in vitro* peptide-HLA binding assays with engineered monoallelic cell lines enabled systematic screening of more than 24,000 neoepitope-HLA combinations, from which 86 validated pairs were identified by immunoprecipitation MS; T-cell receptors targeting selected pairs mediated mutant-specific cytotoxicity in human T cells (80). In parallel, technological innovations continue to improve sensitivity and coverage. Application of trapped ion mobility spectrometry-based MS has expanded benign reference immunopeptidome datasets derived from primary tissues, improving discrimination of tumor-specific antigens (81). Despite these advances, immunopeptidomics remains technically demanding and typically requires large amounts of input material. Emerging microfluidic technologies are beginning to mitigate this limitation, enabling HLA peptide isolation from as few as one million cells (82, 83).

#### 5.2.2 Surface proteomics (surfaceomics)

Surface proteomics, or surfaceomics, refers to the systematic mass spectrometry-based profiling of proteins exposed on the extracellular face of the plasma membrane. Since ExNeoEpitopes are novel peptide sequences embedded within extracellular domains of transmembrane proteins, surfaceomics provides a direct way to confirm that computationally predicted candidates are not only translated but also presented on the tumor cell surface and accessible to antibody-based therapies.

A range of complementary enrichment strategies has been developed to isolate surface proteins for MS analysis. First, chemical biotinylation using cell-impermeable reagents such as sulfo-NHS-biotin labels accessible primary amines on extracellular protein domains, enabling streptavidin pulldown followed by LC-MS/MS identification (84). Second, glycocapture-based cell surface capture (CSC) exploits the widespread N-glycosylation of surface proteins by oxidizing extracellular glycans and enriching labeled glycoproteins via hydrazide chemistry (85). Third, enzyme-mediated proximity labeling provides an alternative strategy in which membrane-localized enzymes such as APEX2 or HRP generate short-lived biotin radicals that tag nearby extracellular proteins *in situ* for subsequent enrichment and MS analysis (86, 87). Fourth, cationic colloidal silica bead coating leverages electrostatic interactions between positively charged silica microbeads and the negatively charged plasma membrane. Following cross-linking with an anionic polymer and cell lysis, the resulting high-density, silica-coated membrane sheets can be separated from intracellular components by density gradient centrifugation, yielding highly enriched plasma membrane fractions while preserving native protein orientation (88, 89). Finally, centrifugation-based membrane fractionation provides a straightforward, label-free strategy. This can be achieved either through differential centrifugation at increasing speeds to separate subcellular compartments and enrich plasma membrane fractions, or through density gradient ultracentrifugation to more precisely resolve plasma membranes from other organellar membranes, followed by proteomic analysis without chemical modification (90, 91).

These approaches have already proven effective for identifying actionable immunotherapy targets. Integration of surface proteomics with transcriptomic data enabled systematic prioritization of CAR-T targets in AML by distinguishing leukemia-specific surface antigens from those expressed on normal hematopoietic cells (92). More recently, structural surfaceomics combining cross-linking MS with CSC identified a tumor-specific conformational epitope of integrin β2, which could be targeted by CAR-T cells to eliminate AML without detectable toxicity to normal cells (93). Large-scale profiling of primary AML samples has further expanded the repertoire of candidate surface antigens, identifying subtype-specific targets with translational potential (94). Complementing these efforts, the Cancer Surfaceome Atlas integrates genomic and functional datasets to systematically annotate cancer-associated surface proteins across tumor types (95).

The same principles can be extended to ExNeoEpitope validation. In this context, a proteogenomic workflow is required in which customized databases incorporating in silico translated splice junction sequences are used to search MS/MS spectra derived from surface-enriched fractions. Detection of junction-spanning peptides provides direct evidence that the aberrant isoform is expressed and exposed at the cell surface. In principle, any surface enrichment method can be paired with such proteogenomic databases to enable systematic discovery of ExNeoEpitopes across tumor samples.

Despite these advances, each enrichment strategy has inherent limitations that must be considered. Labeling-based methods such as sulfo-NHS-biotin and CSC offer high specificity for surface proteins but are prone to false negatives due to dependence on chemical accessibility. Proteins with few exposed lysine residues or lacking N-glycosylation sites may be underrepresented or entirely missed. In contrast, membrane fractionation captures a broader set of membrane-associated proteins but suffers from contamination by intracellular compartments such as the endoplasmic reticulum, Golgi apparatus, and endosomes, leading to potential false positives (96). This is a critical concern for splicing-derived ExNeoEpitope validation, since misfolded or improperly trafficked isoforms may be retained intracellularly yet still appear in membrane fractions.

### 5.3 Long-read and single-cell isoform sequencing

#### 5.3.1 Full-length isoform resolution

Short-read RNA-seq can sensitively detect individual exon-exon junctions, but it cannot reliably reconstruct full-length transcript isoforms or determine which alternative exons co-occur within the same molecule. This limitation has important implications for neoantigen prediction. Without complete isoform information, it is not possible to determine whether a mis-spliced transcript introduces a premature termination codon and is subsequently degraded by NMD, or instead produces a stable, translatable mRNA. Even when transcripts escape NMD, their full exon composition defines the topology of the encoded protein, which ultimately determines whether a splicing-derived neoepitope is exposed on the cell surface or confined to intracellular regions. Resolving full-length isoforms is therefore essential for accurately predicting both protein expression and epitope accessibility.

Long-read sequencing technologies, including Pacific Biosciences (PacBio) SMRT sequencing and Oxford Nanopore platforms (97, 98), address this limitation by directly sequencing full-length cDNA molecules, often spanning 10 kilobases or more, thereby eliminating the need for computational transcript assembly.

A systematic benchmark of Nanopore long-read RNA sequencing across seven human cell lines demonstrated that long-read approaches more robustly capture dominant isoforms than short-read methods, although performance remains influenced by platform-specific trade-offs in accuracy and throughput (99). Consistent with this advantage in disease contexts, full-length transcript profiling of *SF3B1*-mutant CLL using PacBio Iso-Seq revealed novel isoforms and reduced intron retention events that were not detectable with short-read sequencing (100). Building on these capabilities, SNAF incorporates long-read Iso-Seq data to validate predicted ExNeoEpitope-containing isoforms, as exemplified by cross-validation of the SIRPA isoform model in melanoma cell lines (11).

For splicing neoantigen discovery, long-read sequencing provides several key advantages: 1) It confirms that predicted aberrant splice junctions are present within full-length transcripts rather than arising from assembly artifacts. 2) It enables evaluation of whether the resulting isoform encodes a transmembrane protein with a neoepitope exposed on the extracellular surface. 3) By resolving entire transcript molecules, it captures co-occurring splicing events that are not detectable through junction-level analysis alone.

#### 5.3.2 Single-cell isoform resolution

Bulk RNA-seq, even when combined with long-read sequencing, averages isoform expression across all cells in a sample and therefore fails to resolve cell-type-specific splicing. This is a critical limitation in tumors, where malignant cells coexist with normal, stromal, and immune populations. For a splicing-derived neoantigen to be therapeutically actionable, its expression must be restricted to malignant cells and absent from normal counterparts. Conventional short-read single-cell RNA-seq platforms capture only transcript ends and cannot reconstruct full-length isoforms, but recent integration of long-read sequencing with single-cell barcoding has begun to overcome this limitation.

Recent studies highlight the power of this approach across biological contexts. In ovarian cancer, long-read single-cell RNA-seq revealed extensive isoform diversity, identifying over 150,000 isoforms, including more than 50,000 previously unannotated, and uncovering cell-type-specific isoform usage between tumor and mesothelial cells (101). In hematopoietic stem and progenitor cells, integrated short- and long-read single-cell sequencing showed that more than half of expressed genes produce multiple translated isoforms with functional consequences for key regulatory pathways (102). Extending this framework to spliceosome-mutant malignancies, multimodal approaches such as GoT-Splice enable simultaneous profiling of genotype, transcriptome, splicing, and surface proteins at single-cell resolution (103). Application to *SF3B1*-mutant MDS revealed that cryptic splice-site use differs among discrete progenitor states and across lineage transitions, demonstrating that mutation-associated splicing is modulated by cellular context rather than expressed uniformly across the malignant clone (103). AML also contains recurrent tumor-intrinsic splicing programs shared across patients (7), superimposed on cell-state-specific and lineage-associated splicing (104).

An especially important application of single-cell isoform resolution is the identification of neoantigens enriched in cancer stem cell populations. In leukemia, aberrant splice isoforms distinguish leukemia stem cells (LSCs) from both normal hematopoietic stem cells and bulk leukemic cells, and pharmacologic perturbation of splicing preferentially impairs LSC maintenance (105). Since LSCs drive relapse and therapeutic resistance, neoantigens selectively expressed in this compartment represent high-value targets. Long-read single-cell approaches are uniquely suited to identify such candidates, as they can resolve full-length aberrant isoforms within phenotypically defined LSC-enriched populations while simultaneously confirming their absence in normal counterparts from the same sample. More broadly, building single-cell isoform atlases stratified by mutation and cancer type will be essential for defining the specificity landscape required to prioritize neoantigen targets with optimal therapeutic windows.

### 5.4 AI-based structural prediction and *de novo* therapeutic design

Aberrant splice isoforms generate protein sequences that are absent from existing structural databases, making it difficult to predict whether a candidate neoepitope will fold correctly, traffic to the appropriate cellular compartment, or adopt a therapeutically accessible conformation. Recent advances in structure prediction offer partial solutions. AlphaFold 3 extends modeling to multimeric protein-protein and protein-ligand complexes and could, in principle, be used to evaluate the structural context of both peptide-MHC (pMHC) complexes and full-length transmembrane isoforms containing ExNeoEpitopes (106). Building on such structural models, generative design tools are applied to the two major classes of splicing-derived targets: pMHC binders for MHC-presented neopeptides, and epitope-specific antibodies for ExNeoEpitopes.

#### 5.4.1 T cell-targetable splicing neoantigens (peptide-MHC complex)

For splicing-derived peptides presented on MHC-I, recent advances in generative AI have enabled the *de novo* design of proteins that bind specific peptide-MHC complexes with high specificity. Recent work has demonstrated that RFdiffusion-based approaches can generate binders targeting diverse pMHC complexes across multiple HLA alleles, including both tumor-associated antigens and neoantigens (107). These binders can be incorporated into CAR constructs to drive peptide-specific T-cell activation, even in cases where no experimental structure is available and predicted structures are used as input (107). Independent work has validated this strategy using structurally characterized minibinders and TCR-mimetic proteins, with demonstrated tumor cell killing and favorable specificity profiles (108, 109). These advances suggest that splicing-derived neoantigen peptides that are identified computationally and validated by immunopeptidomics could be directly used as inputs for therapeutic binder design, bypassing the need to isolate endogenous TCRs.

#### 5.4.2 B cell-targetable splicing neoantigens (ExNeoEpitopes)

For splicing-derived extracellular neoepitopes (ExNeoEpitopes) embedded in transmembrane proteins, generative design approaches have focused on antibody engineering. A fine-tuned version of RFdiffusion (RFantibody) enables *de novo* design of epitope-specific antibodies entirely in silico (110). Using this framework, nanobodies and scFvs targeting predefined epitopes have been generated with experimentally validated, near-atomic binding accuracy (110). In principle, this approach could be extended to validated ExNeoEpitopes, which enables rapid design of immunotherapeutic modalities including monoclonal antibodies, antibody-drug conjugates, and CAR recognition domains. However, this application critically depends on accurate structural models of the aberrant isoform and correct prediction of extracellular epitope exposure, which remain challenging.

Despite these advances, several key limitations remain across both modalities. Current generative models have been primarily benchmarked on well-characterized antigens, and their performance on structurally atypical splicing-derived peptides, such as those spanning cryptic junctions or containing frameshifted sequences, is unknown. Antibody-antigen docking accuracy is also reduced for key features such as CDR-H3 loop conformations and epitope-paratope interactions, and no available tools account for the altered membrane topology introduced by aberrant splicing events (111). In addition, no existing framework integrates isoform-aware structural modeling, membrane topology prediction, and therapeutic binder design into a unified pipeline. Finally, the immunogenicity and safety of *de novo* designed proteins that diverge from natural antibodies or TCRs require careful evaluation. Addressing these gaps will be essential to enable a fully integrated computational workflow that links aberrant splicing events to clinically actionable therapeutic design.

## 6 Safety considerations

### 6.1 On-target, off-tumor toxicity

A key advantage of splicing-derived neoantigens over conventional tumor-associated antigens is their minimal expression in normal tissues. MHC-presented neopeptides arise from aberrant splice junctions largely restricted to cancer cells, while ExNeoEpitopes originate from tumor-enriched isoforms displayed on the cell surface with minimal expression in normal tissues. This contrasts with shared tumor-associated antigens such as CD33, CD123, and HER2, whose normal-tissue expression necessitates mitigation strategies including antigen-edited hematopoietic stem cell co-delivery and careful dose titration (112, 113). However, this presumed specificity must be rigorously validated. Even low-level expression of a target isoform in normal tissues, which may be undetected by standard RNA-seq, could pose safety risks, particularly for therapies with sustained activity such as CAR-T cells or long-acting antibodies. Comprehensive isoform-level profiling using long-read single-cell RNA-seq and surface proteomics across relevant normal tissues is therefore essential prior to clinical application. For hematologic targets, this includes analysis across discrete stages of hematopoietic differentiation; for solid tumors, it requires evaluation of epithelial, stromal, and neural compartments.

### 6.2 Cross-reactivity assessment

If splicing-derived neoantigen junction sequences share partial homology with peptides from normal tissue proteins, immune responses against the neoepitope may cross-react with these endogenous epitopes and lead to autoimmune toxicity. For T-cell-based strategies, this risk necessitates thorough epitope specificity profiling, including systematic searches of the normal human proteome for peptides with sequence similarity to the target neopeptide presented on the same HLA allele. The *de novo* designed pMHC binders described in Section 5.4 provide a potential advantage, as their peptide-centric recognition allows structure-guided screening against large peptide libraries to identify off-target candidates prior to clinical application (107, 108). For ExNeoEpitope-targeting antibodies and CAR-based approaches, cross-reactivity assessment should extend to surface proteomic profiling of normal tissues to ensure that the epitope is not accessible on any non-malignant cell population.

### 6.3 Safety of combination immunotherapy

The combination strategies described in Section 3.3, although mechanistically well justified, introduce additional safety considerations. For example, combining splicing modulators with checkpoint blockade, as shown by Lu et al. to enhance anti-tumor immunity (54), may increase the risk of immune-related adverse events (irAEs), including autoimmune cytopenias, colitis, hepatitis, and pneumonitis (114). This risk is particularly relevant in vulnerable populations such as older patients with comorbidities or those who have undergone extensive prior treatment. In the post-transplant setting for hematologic malignancies, checkpoint blockade further carries the risk of triggering graft-versus-host disease (115). For spliceosome-targeting agents used to expand neoantigen repertoires, higher doses may disrupt splicing in normal cells, underscoring the need for careful dose optimization to preserve therapeutic selectivity (**Table 2**) (116). While the tumor-restricted nature of splicing-derived neoantigens offers a strong theoretical safeguard against on-target systemic toxicity, the integration of broad immune activation with neoantigen-directed therapies requires rigorous preclinical evaluation to clearly define the therapeutic window for each disease setting and patient population.

## 7 Challenges

### 7.1 Establishing genuine tumor specificity

A key challenge is distinguishing truly tumor-specific splice junctions from the low-level background splicing present in normal tissues. Current filtering strategies often rely on bulk normal reference datasets such as GTEx, which may underrepresent rare cell populations and developmental states. Recent advances in isoform-resolved normal references, particularly long-read atlases of full-length transcripts across GTEx tissues, have greatly strengthened tumor-specificity assessment (45). Extending these references to single-cell resolution remains an important goal, because junctions expressed in rare normal populations can be diluted below detection in bulk data and thereby misclassified as tumor-specific.

### 7.2 Bridging prediction and presentation

A central bottleneck is that only a small fraction of recurrent aberrant splice junctions are expected to yield therapeutically actionable surface ExNeoEpitopes. Importantly, NMD is coupled to translation, so NMD-sensitive transcripts may still generate short-lived products that enter MHC-I antigen-processing pathways; by contrast, a surface ExNeoEpitope generally requires an isoform that avoids or sufficiently escapes NMD to support production of a stable, full-length membrane protein. Candidate prioritization therefore requires evidence that an aberrant junction is tumor-selective and incorporated into a productive full-length isoform that is actively translated; that the resulting protein folds correctly, passes endoplasmic-reticulum quality control, traverses the Golgi, inserts into the plasma membrane with the correct orientation, and displays an extracellular neoepitope that is neither structurally masked nor glycan-shielded.

Surface localization alone, however, does not establish therapeutic relevance. A candidate may be recurrent across patients yet confined within an individual patient to a minor clone, a transient malignant state, or cells that do not propagate the disease. Prioritization should therefore include longitudinal and single-cell assessment of clonal representation across discrete malignant states, persistence through treatment and relapse, and expression in disease-propagating populations, including functionally validated LSCs or LSC-enriched compartments.

Quantitative antigen density, membrane persistence, shedding, internalization kinetics, and antigen-removal mechanisms further determine both therapeutic feasibility and modality selection. Stable surface expression generally favors T-cell-redirecting strategies, whereas efficient endocytosis and lysosomal trafficking favor antibody-drug conjugates; conversely, very low-density targets may be biologically authentic yet unsuitable for direct T-cell redirection. Finally, the targeting reagent must demonstrate isoform-specific recognition without binding relevant normal cells or unrelated proteins. This staged attrition framework distinguishes recurrent RNA events from biologically authentic surface neo-isoforms and, ultimately, from the much smaller subset with genuine translational potential (**Table 4**).

**Table 4:** Validation checkpoints for splicing-derived neoantigen and ExNeoEpitope targets.

Bottleneck	Required evidence	Representative assay	Consequence of failure
Quantitative tumor selectivity	Enrichment over relevant normal cell states	GTEx, single-cell references, primary normal-cell panels	On-target/off-tumor toxicity
Productive full-length transcript	Complete coding architecture and NMD context	Long-read RNA sequencing, RT-PCR/Sanger	No stable protein
Active translation	Translation of the candidate isoform	Ribosome profiling, targeted proteomics	RNA-only candidate
Folding and trafficking	ER/Golgi transit and plasma-membrane insertion	Structural prediction, confocal imaging, live-cell flow	Intracellular retention
Extracellular topology	Neo-sequence exposed on intact cells	Nonpermeabilized flow, topology tags, isoform-specific mAb	Inaccessible epitope
Endogenous specificity	Signal lost after isoform/gene disruption	CRISPR knockout and reference-versus-neo rescue	Artifact or non-isoform-specific reagent
Clonal and LSC persistence	Expression across malignant states and relapse	Longitudinal single-cell/genotype-linked analysis	Incomplete or transient coverage
Antigen density	Sufficient molecules per cell	Calibrated flow cytometry	Inadequate potency
Internalization	Modality-compatible trafficking	pH-sensitive uptake assays	Incorrect modality
Reagent cross-reactivity	Lack of unrelated normal-protein binding	Primary cells, tissue panels, membrane arrays	Antibody-mediated off-target toxicity

Checkpoints a candidate target must clear on the path from an RNA-level prediction to a validated, actionable antigen, with the evidence required, a representative assay, and the consequence of failure for each. Checkpoints progress from transcript- to protein- to target- and modality-level requirements; surface-topology, internalization, and antigen-density checkpoints apply mainly to antibody- and CAR-based approaches against surface ExNeoEpitopes.

### 7.3 Personnel and cross-disciplinary expertise

A practical but often overlooked challenge is the breadth of expertise required. Unlike mutation-based neoantigen discovery, splicing neoantigen research integrates RNA splicing, computational transcriptomics, proteomics, protein structural modeling, and cancer immunotherapy. Because each step depends on the accuracy of the preceding one, errors at any stage can compromise downstream analyses. Continued progress therefore relies on multidisciplinary teams or close collaborations, together with training that bridges RNA biology, immunology, and computational methods.

## 8 Conclusion

Splicing-derived neoantigens define a distinct and expansive class of immunotherapeutic targets that extend beyond conventional mutation-centered frameworks and uncover a previously underexplored layer of tumor specificity within the cancer transcriptome. Arising from diverse mechanisms of splicing dysregulation and often exhibiting tumor-restricted expression with the capacity for recurrence across patients, they offer a compelling and scalable foundation for next-generation cancer immunotherapies. Their translation into the clinic, however, will require careful resolution of key biological constraints, including immune tolerance, impaired antigen presentation, and the immunosuppressive tumor microenvironment. Progress in this area will depend on the continued integration of advanced computational prediction with high-resolution experimental validation, together with rational combination strategies that enhance neoantigen visibility and restore effective immune function. With continued development of these approaches, splicing-derived neoantigens are positioned to play a central role in expanding both the scope and precision of cancer immunotherapy across diverse malignancies.
